# Impact of the COVID-19-pandemic on thrombectomy services in Germany

**DOI:** 10.1186/s42466-020-00090-0

**Published:** 2020-11-23

**Authors:** Steffen Tiedt, Felix J. Bode, Timo Uphaus, Anna Alegiani, Klaus Gröschel, Gabor C. Petzold, for the GSR-ET investigators, Jörg Berrouschot, Tobias Boeckh-Behrens, Georg Bohner, Albrecht Bormann, Michael Braun, Martin Dichgans, Franziska Dorn, Bernd Eckert, Ulrike Ernemann, Jens Fiehler, Christian Gerloff, Gerhard F. Hamann, Karl-Heinz Henn, Fee Keil, Lars Kellert, Christoffer Kraemer, Jan Liman, Alexander Ludolph, Christan H. Nolte, Martina Petersen, Waltraud Pfeilschifter, Sven Poli, Joachim Röther, Eberhard Siebert, Andreas Siedow, Laszlo Solymosi, Florian Stögbauer, Götz Thomalla, Silke Wunderlich, Sarah Zweynert

**Affiliations:** 1 Institute for Stroke and Dementia Research, University Hospital, LMU Munich, Munich, Germany; 2 Department of Neurology, University Hospital, LMU Munich, Munich, Germany; 3 Division of Vascular Neurology, Department of Neurology, University Hospital Bonn, Bonn, Germany; 4 German Center for Neurodegenerative Diseases (DZNE), Bonn, Germany; 5 Department of Neurology, University Medical Center of the Johannes Gutenberg University Mainz, Mainz, Germany; 6 Department of Neurology, University Medical Center Hamburg-Eppendorf, Hamburg, Germany

**Keywords:** Stroke, COVID-19, Thrombectomy, Large vessel occlusion, Outcome

## Abstract

**Background:**

The Coronavirus Disease 2019 (COVID-19) pandemic may have altered emergency workflows established to optimize the outcome of patients with large-vessel occlusion (LVO) stroke.

**Aims:**

We here analyzed workflow time intervals and functional outcomes of LVO patients treated with endovascular thrombectomy (ET) during the COVID-19 pandemic in Germany.

**Methods:**

We compared the frequency, pre- and intrahospital workflow time intervals, rates of reperfusion, and functional outcome of patients admitted from March 1st to May 31st 2020 with patients admitted during the same time interval in 2019 to 12 university and municipal hospitals across Germany (*N* = 795).

**Results:**

The number of LVO patients treated with ET between March to May 2020 was similar when compared to the same interval in 2019. Direct-to-center patients and patients admitted through interhospital transfer in 2020 showed similar pre- and intrahospital workflow time intervals compared to patients admitted in 2019, except for a longer door-to-groin time in patients admitted through interhospital transfer in 2020 (47 min vs 38 min, *p* = 0.005). Rates of reperfusion were not significantly different between 2020 and 2019. Functional outcome at discharge of LVO patients treated in 2020 was not significantly different compared to patients treated in 2019.

**Conclusion:**

Pre- and intrahospital workflows, ET efficacy, and functional outcome of LVO patients treated with ET were not affected during the COVID-19 pandemic in our large cohort from centers across Germany.

## Introduction

The outcome of patients with ischemic stroke and myocardial infarction depends on optimized pre- and intrahospital emergency workflows to minimize the time to reperfusion [1]. The rapidly expanding Coronavirus Disease 2019 (COVID-19) pandemic has caused a reorganization of established workflows to limit spread of the disease [2]. In addition, recent reports have also indicated that patients with acute stroke or myocardial infarction might resist or delay seeking help because of fear of COVID-19 [3, 4], raising concerns about worse outcomes of these conditions during the pandemic. Hence, monitoring of time-to-treatment intervals and disease outcomes during the pandemic is highly relevant for policymakers as it allows to assess and act upon the potential collateral effect of implemented COVID-19-related algorithms in the emergency sector. Here, we aimed to analyze workflow time intervals and functional outcomes of LVO patients treated with endovascular thrombectomy (ET) during the COVID-19 pandemic in a large German cohort.

## Methods

The data that support our findings are available from the corresponding author upon reasonable request.

Total numbers of patients treated with ET, patients with ischemic stroke, and patients with transient ischemic attacks were retrieved from hospital databases using the OPS code 8–836.80 and the ICD-10 codes I63 and G45, respectively. Detailed clinical data of this database were available from 795 patients treated with ET (87%) from the German Stroke Registry Endovascular Treatment (NCT03356392) [5], an ongoing, open-label, academic, prospective, multicenter registry in Germany. The main inclusion criteria were a diagnosis of acute ischemic stroke due to LVO, initiation of ET, admission between March 1st and May 31st 2020 or the same period in 2019, and age > 18 years without any exclusion criteria. Patients were recruited by 12 centers (7 university hospitals, 5 municipal hospitals) distributed across Germany. Baseline characteristics, pre- and intrahospital workflow times and rates of reperfusion of the study sample were compared using Mann–Whitney and Fisher’s exact tests. Successful reperfusion was defined as a modified Thrombolysis in Cerebral Infarction (mTICI) scale score of 2b or 3 (the mTICI score was graded locally). To determine associations with the modified Rankin Scale (mRS) score (as dependent variable), we used ordinal multivariable logistic regression adjusting for potential baseline confounder variables (age, sex, National Institutes of Health Stroke Scale (NIHSS) score upon admission, anterior/posterior circulation occlusion, intravenous alteplase use). The cumulative incidence of regional COVID-19 cases was retrieved from the Robert Koch Institute for the interval between March 1st and May 17th 2020, thus covering the exponential growth phase of COVID-19-positive cases in Germany defined by the basic reproductive number R0 > 1.0. The study was conducted in accordance with the Declaration of Helsinki and was centrally approved by the Institutional Review Board of the Ludwig-Maximilians-Universität Munich (protocol No. 689–15) and from local institutional review boards according to local regulations.

## Results

**Fig. 1:**
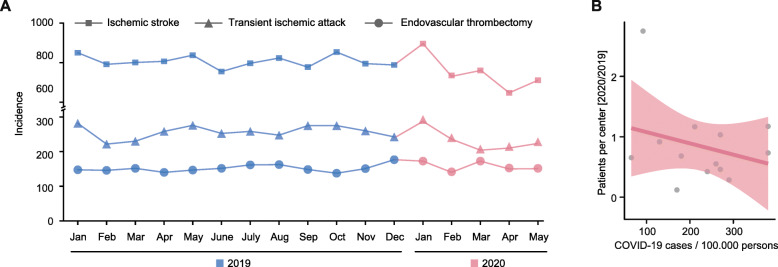
Impact of the COVID-19 pandemic on the number of ET-treated LVO stroke patients in Germany. **a** The number of patients treated with ET showed no major trend between January 2019 and May 2020. The numbers of patients admitted with ischemic stroke and transient ischemic attack showed a slight reduction during the pandemic months (March to May 2020) when compared with the previous months in 2019 and 2020. **b** The ratio of patient numbers per center between 2020 and 2019 was not associated with the regional cumulative incidence of COVID-19 (univariable linear regression model). ET, endovascular treatment

The number of patients treated with ET did not show major trends between January 2019 and May 2020 that would have indicated a potential reduction during the COVID-19 pandemic from March to May 2020 (Fig. 1a), while the numbers of patients with ischemic stroke and transient ischemic attack were slightly reduced during the pandemic. The ratio of patients admitted in 2019 and those admitted in 2020 per center was not associated with the regional cumulative incidence of COVID-19, which ranged from 65 to 380 cases per 100.000 residents (*p* = 0.36, Fig. 1b).

Patients admitted from March to May 2020 and the same time interval in 2019 (*N* = 795) were similar with regard to age, sex, comorbidities, the pre-stroke mRS, the Alberta Stroke Program Early CT score, and the NIHSS score upon admission. While the rate of patients admitted through interhospital transfer was not significantly different between 2019 and 2020, we observed a trend for a higher rate of patients treated with intravenous alteplase in 2020 (Table 1). Three patients treated with ET had a positive PCR test result for SARS-CoV-2.

**Table 1:** Baseline patient and treatment characteristics 2019 and 2020

Characteristics	GSR-ET March to May *N* = 795		***P***
	2019	2020	
Age, median (IQR) [years]	77 (66–83)	76 (65–82)	0.717
Female, % (n)	54.5 (234)	51.0 (186)	0.319
Medical history, % (n)			
Hypertension	79.8 (339)	79.4 (285)	0.929
Diabetes mellitus	25.4 (108)	24.4 (88)	0.804
Atrial fibrillation	42.8 (181)	40.4 (138)	0.555
Pre-stroke mRS score > 1, % (n)	22.9 (96)	19.5 (66)	0.285
Baseline NIHSS score, median (IQR)	15 (9–18)	14 (9–18)	0.799
ASPECTS, median (IQR)	9 (8–10)	9 (8–10)	0.911
External, % (n)	43.8 (188)	38.5 (141)	0.149
Anterior circulation occlusion, % (n)	86.6 (361)	89.5 (307)	0.222
Intravenous alteplase treatment, % (n)	43.4 (185)	50.4 (181)	0.053
General anesthesia, % (n)	70.3 (289)	70.2 (240)	1

*IQR* Interquartile range, *mRS* Modified Rankin Scale, *NIHSS* National Institutes of Health Stroke Scale, *ASPECTS* Alberta Stroke Program Early CT Score

**Fig. 2:**
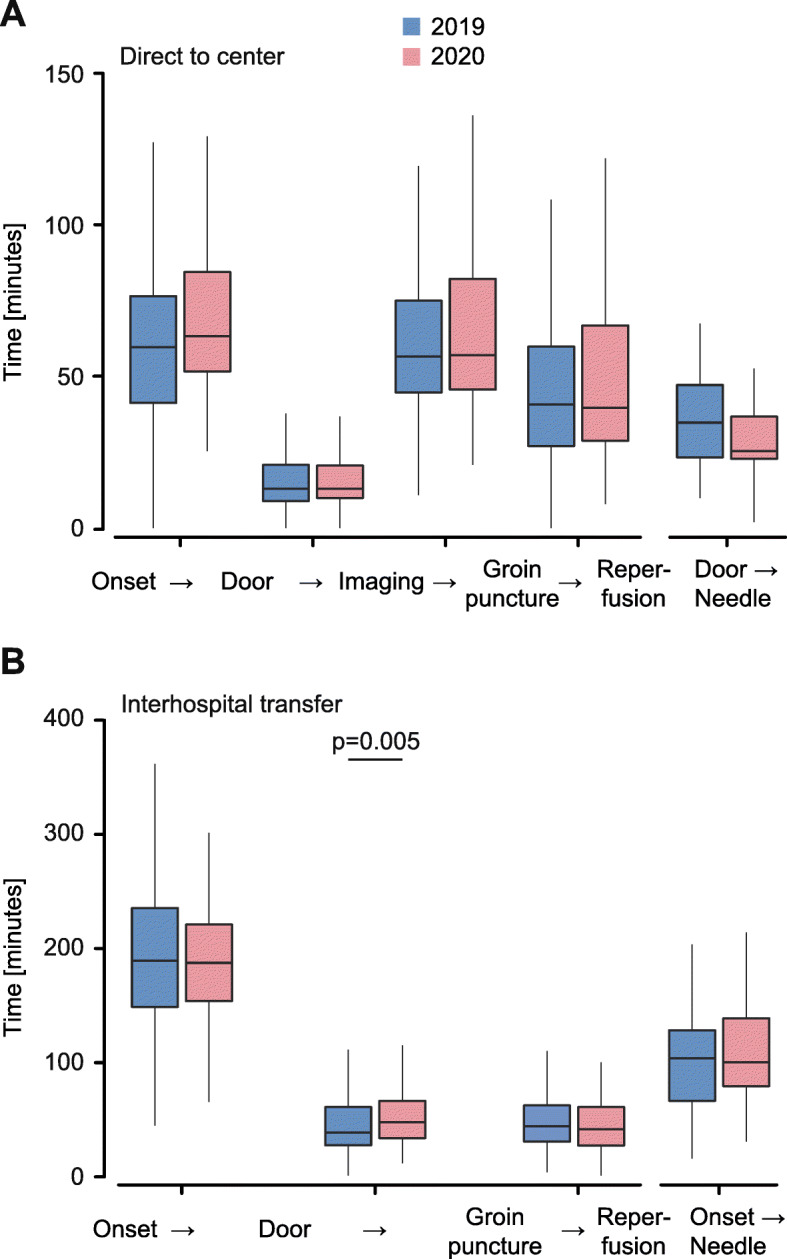
Impact of the COVID-19 pandemic on pre- and intrahospital workflow time times of LVO patients treated with ET. **a** Workflow time intervals were similar for direct to center patients between 2020 and 2019 (Mann-Whitney test). **b** Workflow time intervals were similar for patients undergoing interhospital transfer between 2020 and 2019 except for a longer door-to-groin time in 2020 (Mann-Whitney tets). ET, endovascular treatment; LVO, large vessel occlusion

**Fig. 3:**
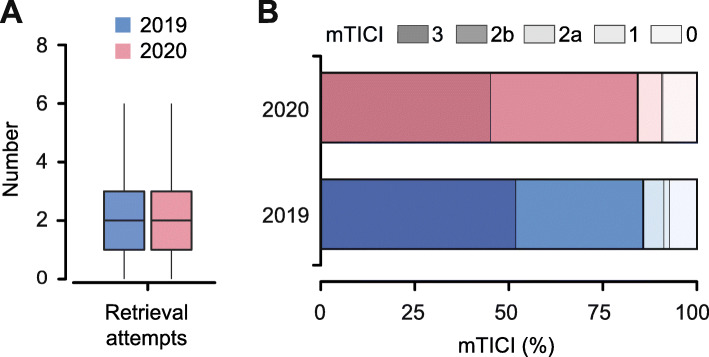
Impact of the COVID-19 pandemic on efficacy measures of ET. **a** The number of retrieval attempts needed until successful reperfusion was not different between patients with anterior circulation stroke treated in March–May 2020 when compared with the same period in 2019 (Mann-Whitney test). **b** The rate of successful reperfusion was not different between 2020 and 2019 (Fisher’s exact test). ET, endovascular treatment; mTICI, modified Thrombolysis in Cerebral Infarction

Direct-to-center patients admitted in 2020 showed similar pre- and intrahospital workflow time intervals compared to patients admitted in 2019 (Fig. 2a). Patients admitted through interhospital transfer in 2020 also showed similar workflow time intervals compared to patients in 2019, except for a longer door-to-groin puncture time in 2020 (47 min vs 38 min, *p* = 0.005, Fig. 2b). The frequency of general anesthesia was similar in 2020 compared to 2019 (Table 1). ET efficacy measures, such as the number of retrieval attempts (Fig. 3a) and the rate of successful reperfusion, did not differ between patients in 2020 and 2019 (Fig. 3b).

**Fig. 4:**
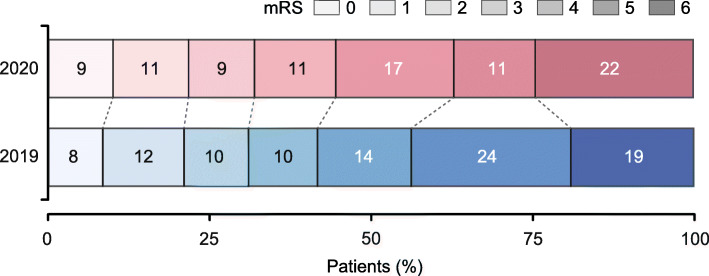
Impact of the COVID-19 pandemic on functional outcome after ET. Functional outcome at discharge of patients treated in March–May was not different between 2020 and 2019 in analysis adjusting for potential baseline confounders (multivariable logistic regression analysis). mRS, modified Rankin Scale; ET, endovascular treatment

Functional outcome at discharge was not different between 2020 and 2019 after adjustment for potential baseline confounders (adjusted odds ratio, 1.00 [95% CI, 0.77–1.31]; Fig. 4).

## Discussion

In this study, based on data from almost 800 patients, we found no changes in workflow time intervals, ET efficacy, and functional outcomes in LVO patients treated with ET during the COVID-19 pandemic in Germany when compared to 2019.

Our study needs to be put into perspective and compared to recent reports on stroke admission numbers and workflow time intervals from other countries and regions. While we found a slight reduction of patients admitted with ischemic stroke and transient ischemic attack but no change in patients treated with ET, reductions in stroke patient numbers during the COVID-19 pandemic were also found in China and Spain when all stroke admissions were analyzed [6, 7], and in France when patients treated with ET were analyzed [8]. Moreover, longer workflow times were found in France [8] but not in Spain [7]. In contrast, in our analysis the majority of workflow times were similar between 2020 and 2019 except for a longer door-to-groin puncture time in patients admitted through interhospital transfer in 2020. This may potentially be related to a higher level of uncertainty regarding the infectious status of patients from other hospitals, although we have no data to support this interpretation. We hypothesize that the differences between these findings and our results are likely related to a combination of epidemiological and health system-related factors, which may include: i) differences in the COVID-19 incidence between regions and countries, ii) different levels of lockdown measures between countries, iii) the pre-pandemic state of these health care systems, and iv) the ability of healthcare providers and policymakers to prepare for the impact of the pandemic. Integrating these data from different countries might inform policymakers and health care providers on how to react adequately to future pandemics, or a potential ‘second wave’ of the current pandemic, while maintaining optimized emergency workflows for patients with acute ischemic diseases. In our cohort, three patients were tested positive for SARS-CoV-2, however, we note that routine testing was not implemented in most hospitals during that time, indicating that the actual number might be higher.

The strengths of our study include the large sample size and multi-center nature of our study, as well the report of functional outcome measures in addition to procedural times. Importantly, our data were collected from both university and municipal hospitals and from regions differently affected by the COVID-19 pandemic. Our study is limited in its observational character, and we thus cannot rule out residual confounding. Moreover, our data were obtained from stroke centers with highly standardized prehospital and intrahospital algorithms, and may thus not be generalizable to lower-volume or non-specialized hospitals.

We conclude that pre- and intrahospital ET workflows, ET efficacy, and functional outcome of LVO patients were not affected during the COVID-19 pandemic in our large German cohort. Close monitoring of workflow intervals remains important to secure optimized care of hyperacute emergencies during the pandemic.

## Data Availability

The datasets used and/or analyzed in the current study are available from the corresponding author on reasonable request.

## Abbreviations

LVO: Large-vessel occlusion
COVID-19: Coronavirus Disease 2019
ET: Endovascular thrombectomy
mRS: Modified Rankin Scale
NIHSS: National Institutes of Health Stroke Scale
ASPECTS: Alberta Stroke Program Early CT Score
mTICI: Modified Thrombolysis in Cerebral Infarction
